# Factors Influencing the Adoption of Shared Autonomous Vehicles

**DOI:** 10.3390/ijerph17134868

**Published:** 2020-07-06

**Authors:** Kum Fai Yuen, Do Thi Khanh Huyen, Xueqin Wang, Guanqiu Qi

**Affiliations:** 1 School of Civil and Environmental Engineering, Nanyang Technological University, Singapore 639798, Singapore; kumfai.yuen@ntu.edu.sg; 2 Department of International Logistics, Chung-Ang University, Seoul 06974, Korea; huyendo21@cau.ac.kr (D.T.K.H.); wang1072@e.ntu.edu.sg (X.W.)

**Keywords:** shared autonomous vehicles, unified theory of acceptance and use of technology, theory of planned behaviour, adoption

## Abstract

Shared autonomous vehicles (SAVs), which have several potential benefits, are an emerging innovative technology in the market. However, the successful operation of SAVs largely depends on the extent of travellers’ intention to adopt them. This study aims to analyse the factors that influence the adoption of SAVs by integrating two theoretical perspectives: the unified theory of acceptance and use of technology 2 (UTAUT2) and the theory of planned behaviour (TPB). A valid survey sample of 268 participants in Da Nang, Vietnam was collected. Subsequently, structural equation modelling was deployed to test the research model. The results indicate that the five dimensions of UTUAT2: performance expectation, effort expectation, habit, price value and hedonic motivation, are mediated by the attitudes toward using SAVs. Further, the TPB constructs, namely attitude, subject norm, perceived behavioural control, along with its perceived facilitating conditions, are all effective predictors of intention to use SAVs. The findings of this study can serve as a crucial resource for transport operators and the government to enhance transportation services and policies.

## 1. Introduction

Shared autonomous vehicles (SAVs) are considered a technology that can offer solutions to transportation problems by improving passengers’ safety and quality of life while reducing traffic congestion and pollution [1]. SAVs are a form of self-driving transportation that provides on-demand services and non-fixed routes for passengers. Although the acceptance of self-driving vehicles in the near future seems to be a difficult task, previous studies [2,3,4] have demonstrated that autonomous vehicle (AV) technologies will become more widespread in the international market by the end of the decade due to their potential benefits.

There are many benefits to the deployment of SAVs. For instance, SAVs can enhance traveller safety by reducing crashes caused by human error (i.e., alcohol, exhaustion, loss of control) [2]. Moreover, according to a previous study [5], a traditional taxi service can only facilitate point-to-point travelling for an individual passenger or a group of passengers. Vacant taxis usually cruise along urban roads picking up customers. This not only leads to heavy traffic, especially during peak times, but is also an economic burden. In contrast, SAVs are fleet services with dynamic ridesharing (DRS), which can transport multiple customers with the same pick-up location and nearly the same drop-off destination in the same vehicle [2,6]. This can create cost efficiency (i.e., operation cost, fare, labour costs) and reduce the number of vehicles on the road, leading to reduced traffic congestion, fuel consumption and pollution reduction. Lastly, a fully automated vehicle can facilitate smoother braking, speed adjustment and optimize routing, which can improve the quality of public transportation [2]. Such an innovative technology can also enable travellers to utilize their commute time productively, e.g., reading, working, or watching a movie [3,7].

Despite SAVs’ great benefits, their adoption in the future is uncertain due to various consumer concerns such as security, safety, legal issues and privacy [8]. Additionally, since the SAV system is an emerging transportation mode that differs substantially from existing modes of public transport, a significant number of people may be apprehensive about utilizing SAVs. Hence, it is important to understand the apprehensions of consumers who will potentially use and accept SAVs [6,9].

In recent years, many studies have been conducted about consumers’ perceptions toward AV technology in the private vehicle market but relatively fewer studies have been focused on the adoption of SAVs in the public transport market [10]. Moreover, most research has only analysed the socio-demographic and commute characteristics of users with regards to the adoption of driverless vehicles [11,12,13]. Thus, this gap regarding self-driving vehicles in previous literature reviews can be bridged by analysing psychological factors in theories, e.g., attitudes, subject norms and behaviour control. According to Jing et al. [14], the application of theory-based models can provide stronger predictors and explanations about the determinants of SAV adoption intention. For instance, Zhang et al. [15] applied a theoretical framework, the technology acceptance model (TAM), to explore and predict the intention to use AVs. The TAM theory demonstrated that users’ perception of the usefulness and perceived ease of use of automated systems has a positive influence on AV adoption. Jing et al. [14] applied the theory of planned behaviour, which suggests that variables, such as attitude, subject norms, and perceived behavioural control, positively influence travellers’ intention towards using AVs and SAVs.

This study aims to precisely predict user adoption of SAVs by proposing and synthesizing two theoretical perspectives: the unified theory of acceptance and use of technology 2 (UTAUT2) and the theory of planned behaviour (TPB). The motivation for applying these two theoretical frameworks is that they propose unique aspects to explain travellers’ adoption of vehicles that utilise new technologies and emphasize key social elements in using innovative vehicles like SAVs [8,9]. The UTAUT2 can help to effectively analyse adoption behaviour towards novel technologies, such as SAVs, using salient predictor variables related to expectations, values, habits and enjoyment [16].The TPB can help to examine SAV adoption from the perspective of attitudes, subject norms and perceived behavioural control [14]. These psychological factors from theoretical frameworks can provide more systematic prediction of user adoption behaviour and offer deeper insights.

The contribution and novelty of this study are that it applies and synthesises theoretical insights from UTAUT2 and TPB to examine the factors influencing user adoption of SAVs. In particular, this study examines the nomological network which concerns the direct and indirect relationships between the factors. The results obtained from this study can also guide policy formulation, allowing the government to focus on allocating resources on addressing pertinent factors that have strong influences on user adoption of SAVs. Methodological wise, since this study involves the examination of the correlations between latent factors which are multidimensional and involves multiple endogenous (i.e., dependent) factors, structural equation modelling is viewed to be the most suitable method as compared to regression analysis, which is more suited for the examination of manifest variables and only one dependent variable.

The remainder of this article is organized as follows: Section 2 describes the theoretical framework and research hypotheses used to analyse the factors influencing users’ adoption of SAVs. Section 3 presents the applied methodology, including survey questionnaire design and the analysis of data collected from citizens in Da Nang, Vietnam. Vietnam is selected because most SAV or AV adoption research is conducted in more advanced or developed countries. Therefore, it will be valuable to examine this research topic in Vietnam. The results are then presented and discussed in Section 4 using structural equation modelling. Finally, Section 5 provides the conclusions, which includes theory and policy implications to improve users’ adoption of SAVs.

## 2. Literature Review

### 2.1. Theories, Theoretical Models and Hypotheses

This study utilized two theories, the TPB and the UTAUT2, to examine the factors influencing users’ adoption of SAVs and clarify the relationship between these factors. Table 1 summarises the representative constructs and contributions of these theories.

**Figure 1:**
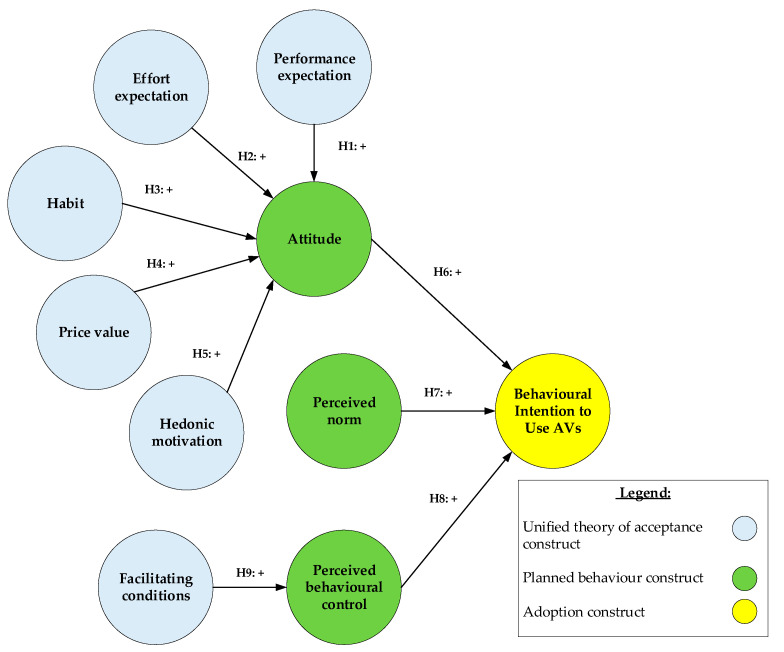
The theoretical model. All constructs are positively worded for ease of reference.

A theoretical model that identifies the latent constructs that influence user adoption of SAVs and specifies their interrelationship was developed (Figure 1). First, according to the TPB [17], consumers’ adoption of new technologies, such as SAVs, can be explained by three critical psychological constructs: attitudes, subject norms and perceived behavioural control.

Second, according to UTUAT2, a comprehensive theoretical model that synthesizes its predecessors, such as the unified theory of acceptance and use of technology (UTUAT), the theory of reasoned action (TRA), diffusion of innovation (DOI) theory and the TAM, attitudes are the key factors influencing consumers’ adoption of new technology [18,19]. Furthermore, attitudes are influenced by five components: performance expectation, effort expectation, habit, price value and hedonic motivation, which can positively influence attitude formation towards SAV adoption by consumers. Further, Hung et al. [20] proposed that perceived behavioural control is influenced by facilitating conditions. In this study, the hypotheses of these constructs will be explicitly argued to derive comprehensive insights into the factors influencing SAV adoption intention.

#### 2.1.1. Determinants of Users’ Attitude Towards SAV Adoption

This section discusses the five components of UTAUT 2 ($H_{1}–H_{5}$) and their influence on users’ attitudes towards using SAVs. Attitudes regarding the use of a product or service, such as a SAV, depends on the users’ perception of its utility. Moreover, attitudes can be affected by several factors such as culture, economy, emotions and perceived value [21].

In recent decades, various behavioural theories, such as the TRA, TPB, TAM, DOI and UTAUT [22], have been accepted and applied to analyse user behaviour towards new technology adoption. In this study, the dimensions influencing user attitudes towards SAVs can be identified using UTAUT2, which is a predictive framework that includes the main constructs that determine consumer intention to use SAVs. These constructs are performance expectation, effort expectation, habit, price value and hedonic motivation [23].

The first determinant of SAV is performance expectation. It is conceptualized as the benefits that the consumers can obtain when they apply the new technology products or new technology services [24]. Davis et al. [25] concluded that performance expectation is the most important determinant of users’ intention to use new technologies. SAVs can offer several advantages and utilities to consumers in their daily lives, listed as follows.: (1) SAVs offer increased safety. According to the U.S. National Highway Traffic Safety Administration [26], 90% of all accidents are caused by driver error [27], and autonomous vehicles can potentially reduce these human failings; (2) SAVs can save service time and travel costs by providing services on less extensively used roadways. Moreover, SAVs can connect a variety of travellers with similar origins and destinations [4,28]; (3) SAVs can reduce emissions and congestion. There is some evidence that advanced systems in an AV, such as adaptive cruise control (ACC), can optimize traffic flow, enhancing fuel consumption and reducing CO_2_ emissions and congestion on roads [3,10]. Thus, these benefits can affect users’ attitudes towards SAV adoption, contributing to the economy, emotions and value factors. Therefore, the following hypothesis was proposed:

**Hypothesis 1** **(H1).** *Performance expectation has a positive effect on consumer attitudes towards the use of shared autonomous vehicles.*

The second determinant is effort expectation, which is conceptualized as the extent of the ease associated with the use of a new technology system [24]. This concept is similar to the ‘perceived ease of use’ factor in the TAM and the ‘complexity’ factor in the DOI theory [22]. Several studies have empirically supported that perceived ease of use refers to the degree of the consumers’ expectations that using a system must be free of effort and not difficult [23,24,25]. Meanwhile, Rogers [29] defined complexity as how difficult it is to use and understand an innovative technology.

SAVs have some unique features that can be designed to reduce consumers’ effort expectation. For instance, the application for booking SAVs can be simplified. Human interaction with the SAVs could also be designed to be intuitive and logical. Demonstration videos can be created to train and educate consumers on booking and interacting with SAVs. These would save time and money for consumers and improve their attitude towards SAVs. Thus, the following hypothesis was proposed:

**Hypothesis 2** **(H2).** *Effort expectation has a positive effect on customer attitudes towards the use of shared autonomous vehicles.*

The third determinant is habit. Habit is the extent to which people tend to automatically perform behaviours developed by learning [24,30]. Kim et al. [31] define habit as past use that was found to be a strong predictor of subsequent use. In line with this assumption, in order to measure habit, previous studies have embraced several proxies for habit such as past behaviour, reflexes and individual experience [22,30]. Past behaviour is viewed as prior behaviour and is a surrogate measure. Reflexes represent behavioural sequences that do not need to be learned. Individual experience is described as the experience that users gain from stable routines, norms and habits in using technology. This diminishes the need for discussions, coordination or effortful decision making.

It was found that users who commute often to their destinations are more likely to use SAVs, while users who do not use public transportation tend to disapprove shared vehicles [9,10]. Additionally, previous use that can form habits enhances interactions and familiarity with novel technologies and influences consumers’ future technology use [24]. If consumers use SAVs for an extended period, then their SAV use habit will be stored in their conscious mind and be automatically established. In contrast, consumers who have not used SAVs before would need more time and effort to learn and familiarize themselves with SAVs. This can create inconvenience and resistance, which could lead to negative attitudes towards using SAVs. This finding is also consistent with previous research regarding novel car adoption [32]. Thus, in this context, the following hypothesis was proposed:

**Hypothesis 3** **(H3).** *Habit of using novel technologies has a positive effect on customer attitudes towards the use of shared autonomous vehicles.*

The fourth determinant is price value, which is described as the trade-off between benefits that customers derive from using SAVs and the monetary cost of using such technology systems [24,33]. Previous literature reviews have argued that monetary value has a crucial impact on consumers’ use of technology products or services. In line with this assumption, J.D. Power and Associates [34] found that 79% of consumers were willing to bear an additional cost of US$250 to purchase a vehicle equipped with autonomous technologies. However, this result dropped to 55% when the purchase price was raised by $300. Price value is considered positive when consumers perceive that the advantages and utility derived are greater than the monetary cost paid. Likewise, users who have a greater appreciation for the value of SAVs would be willing to pay to use such technologies. Additionally, Haboucha et al. [9] postulated that price plays a salient role in encouraging individuals to use SAVs. Thus, the following hypothesis was proposed:

**Hypothesis 4** **(H4).** *Price value has a positive effect on customer attitudes towards the use of shared autonomous vehicles.*

The fifth determinant is hedonic motivation, which is defined as the fun or enjoyment that users derive from using SAVs. Many previous studies have demonstrated that hedonic motivation is a significant determinant in shaping consumers’ intention to use new technologies [22,23,24]. Venkatesh et al. [24] and Becker and Axhausen [12] suggested that for both people who seek excitement and adventure and people who seek innovativeness and novelty tend to use new technology early. However, they also noticed that, in the primary stage of using new technology products, users could find the attractiveness of the novelty more easily. Later, these users might get bored and seek to gain efficiency from the technology products rather than novelty. Thus, the degree of hedonic motivation that contributes to users’ technology use diminishes with an increase in proficiency. Bay [35] suggested that consumers’ perceived enjoyment and entertainment while using technology could have a possible impact on their attitudes towards using self-driving vehicles. Therefore, the following hypothesis was proposed:

**Hypothesis 5** **(H5).** *Hedonic motivation has a positive effect on customer attitudes towards the use of shared autonomous vehicles.*

#### 2.1.2. Determinants of Users’ Intention to Use SAVs

Previous research shows that the TPB, an extension of the TRA, is the most popular model used to examine user adoption of an innovation. The TPB postulates that behavioural intention is governed by three main constructs: attitude, subjective norms and perceived behavioural control.

In this study, these three main constructs and the salient characteristic of innovation, facilitating conditions, were utilised to examine consumers’ adoption of SAVs. The first determinant of users’ SAV adoption intention is attitude, which refers to attitudinal beliefs that lead behaviour to a certain outcome, influenced by the assessment of the desirability of that outcome [36,37]. Previous innovation literature has stated that attitudes towards behavioural intentions are influenced by three salient attitudinal beliefs: relative advantage, complexity and compatibility, which are defined by innovation diffusion benefits [32,36,37]. By analysing these beliefs, the relationships between constructs can be explained more satisfactorily and become clearer [32,38]. Relative advantage refers to the degree to which an innovation is better than its precursor and includes factors such as enhancement and performance benefits. Complexity refers to the degree to which an innovation is perceived to be difficult to understand, learn and operate. Compatibility refers to the degree to which an innovation is perceived as being in line with a potential adopter’s existing values, previous experiences and current needs [29]. Thus, if SAVs were perceived as possessing greater performance benefits (i.e., safety, time and cost savings), effortless (i.e., ease of use, simple), compatible (i.e., lifestyle, habit) and hedonic (i.e., fun, enjoyable) than conventional public transportation, users would be more likely adopt SAVs.

Additionally, the positive effect of attitudes on the intention to adopt new innovative vehicles has been supported by several studies [35,39,40]. Therefore, the following hypothesis proposed:

**Hypothesis 6** **(H6).** *Attitude has a positive effect on customers’ adoption of shared autonomous vehicles.*

The second determinant is subjective norms, which refers to the extent to which an individual perceives that important people or significant reference groups want them to perform or avoid performing a certain behaviour [41]. The more that individuals consider that influential referents (i.e., relations, friends and colleagues) think they should engage in a certain behaviour, the more they comply with adopting this behaviour. Indeed, the perceptions of people who are important to an individual has a salient influence on whether consumers engage in a particular intention or not. Several studies have indicated that subject norms play an important role in influencing behavioural intention [18,35,36]. Moreover, Petschnig et al. [40] stated that subject norms can significantly influence the motivation to adopt an innovative car. Based on the above discussion, the following hypothesis was presented:

**Hypothesis 7** **(H7).** *Subject norm has a positive effect on customers’ adoption of shared autonomous vehicles.*

The third determinant is perceived behavioural control, which is defined as individuals’ perception of ease or difficulty in performing a behaviour of interest [41]. Perceived behaviour control also refers to an individual’s perception of the presence and absence of requisite resources and opportunities. As discussed earlier, when individuals possess substantial resources (i.e., money, time, technology) to make full use of an innovation, they will have a higher level of perceived behavioural control, which faciliates the behavioural intention. Additionally, individuals’ adoption intention may be also positively influenced by whether they recognize the use of SAVs as being more simple and cheaper than existing rail or taxi services. In contrast, a scarcity of necessary resources may inhibit adoption intention [37]. Further, Bay [35] demonstrated that, in the innovation adoption context, perceived behavioural control has a significant influence on the adoption of self-driving cars. Hence, the following hypothesis was constructed:

**Hypothesis 8** **(H8).** *Perceived behavioural control has a positive effect on customers’ adoption of shared autonomous vehicles.*

#### 2.1.3. Determinants of Perceived Behavioural Control

This study proposes that perceived behavioural control is influenced by facilitating conditions. Venkatesh et al. [42], as cited by Huang and Kao [22] and Alalwan et al. [23], defined facilitating conditions as the extent to which an individual perceives that adequate organizational or governmental support and technical infrastructure exists to support the use of a new technology system.

Previous studies have indicated that facilitating conditions play a significant role in usage behaviour among users [22,23,24,43]. Indeed, customers could be motivated to continue using an innovation if they get a certain level of technological support, knowledge and resources (e.g., availability of customer service, online tutorials, time, money). In fact, people who consider a new service as being compatible with technology that they have already used before as well as easy to use and requiring minimal effort will be more likely to opt for SAV systems.

Accordingly, government support can play an intervention and leadership role in the diffusion of innovation. The adoption of innovative applications will be considered as more feasible when individuals perceive a greater level of government support [38,44]. Previous research has also demonstrated that perceived behavioural control is affected by some beliefs, which includes the users’ facilitating conditions when they use innovations [18,19]. Thus, the perception regarding the favourability of technological infrastructure could influence the perceived behavioural control of customers in adopting SAVs.

**Hypothesis 9** **(H9).** *Facilitating conditions have a positive effect on customers’ perceived behavioural control of shared autonomous vehicles.*

## 3. Materials and Methods

As discussed above, Figure 1 displays the conceptual model hypotheses based on the UTUAT2 and TPB frameworks. The UTUAT2 proposes five main predictors: performance expectation, effort expectation, habit, price value and hedonic motivation, while the TPB proposes four main constructs: attitudes, subject norms, perceived behavioural control and the underlying belief structure known as facilitating conditions to interpret users’ behavioural intention towards adopting SAVs.

### 3.1. Measurement Items

This study involves the examination of latent variables, namely performance expectation, effort expectation, habit, price value, hedonic motivation, attitude, subject norms, perceived behavioural control and intention to adopt SAVs. These latent variables are unobservable, so measurement items were generated to operationalize each variable.

### 3.2. Survey Design and Administration

The questionnaire includes three parts. The first part briefly introduces the objective of the research and provides a definition of SAVs to provide respondents with general information when completing the survey questionnaire. Specifically, SAVs were described as driverless taxis or ridesharing vehicles equipped with cameras, sensors and integrated light detection and ranging (LiDAR) systems that helps to identify obstacles, avoid collisions and recognize objects and people on the road while travelling. SAVs are expected to offer travellers potential benefits such as reduced risk of crashes, fuel consumption and traffic congestion. All the respondents’ identities were committed to remain undisclosed under any circumstances. The participants were guided to answer the questions using a five-point rating scale, with 1 being ‘strongly disagree’ and 5 being ‘strongly agree’. The second part of the questionnaire includes answering questions regarding the respondents’ demographic characteristics, i.e., gender, age, monthly income and driving experience. The third part of the questionnaire presents the measurement indicators shown in Table 2.

The questionnaire was targeted towards residents in Da Nang, Vietnam. The English-version questionannire was first developed and then translated into Vietnamese and verified by an editor to ensure that there were no errors and opportunities to misunderstand the indicators. Next, the Vietnamese version was translated into English by another translator to ensure translation equivalence. The questionnaire was then distrubuted online via a Google survey form. A QR code was generated and distributed at three shopping centres: Vincom Centre, Indochina Riverside Mall or Parkson Ving Trung Plaza.

A representative was stationed at the entrance of a shopping centre once every fortnight to approach passersby at random. Thereafter, they were invited to complete the online survey questionnaire. No incentives were given for the completion of the questionaire. A total of 268 completed survey questionnaires were collected between 28 November 2019 and 7 April 2020 in Da Nang Vietnam. All these questionnaires were administered online.

### 3.3. Demographics of Respondents

Table 3 presents the demographic characteristics of the 268 participants in this survey. Among the survey participants, 47% were males while 53% were females. Regarding age, the sample had the highest distribution of respondents aged 18 to 35 years old (35.1%) while 25% of the respondents were aged 36 to 45 years old, the remaining 20.5% and 19.4% accounted for respondents aged more than 45 years old and under 18 years old, respectively.

Additionally, a majority of the respondents reported a monthly income ranging between 6 million VND and 12 million VND (34.4%). The remainder reported monthly income ranges as follows: 13 million to 20 million VND (27.4%), less than 6 million VND (25.6%) and more than 20 million VND (12.6%).

## 4. Results and Discussion

### 4.1. Measurement Model Analysis

A confirmatory factor analysis was conducted to evaluate the goodness-of-fit of the measurement model, as well as the reliability and validity of the indicators. The confirmatory factor analysis is to determine whether the indicators of a construct are consistent with researchers’ understanding of a construct. Its purpose is to determine if the data fit the measurement model proposed in Table 2. Table 4 presents the standardized factor loading (λ), the average variance extracted (AVE) and the composite reliability (CR) for each construct.

Regarding the model fit’s indices, the chi-square fit index (*χ^2^*) of the structural model was 1070.71 and the degree of freedom(*df*) was 449. However, the chi-square fit index tends to be affected by various factors, such as sample size, distribution of variables, lack of fit, etc., so it is not very useful in determining whether a model should be accepted or rejected [50]. The remaining fit indices, i.e., the comparative fit index (CFI) and the Tucker-Lewis fit index (TLI), are above 0.9 and the root mean square error of approximation (RMSEA) is below the 0.08 threshold. These values satisfy the cut-off for a good fitting model, as stated by Hu and Bentler [51]. Thus, the measurement model is considered to have a good fit.

Regarding the reliability of the measurement model, the standardized factor loadings (*λ***)** were examined to evaluate the correlation between the indexes, variables, and the CR values of the variables. As shown in Table 4, overall, all standardized factor loadings and CR values were greater than the recommended values of 0.5 and 0.7 [52,53]. This suggests that all the observed variables have high reliability and power explanation for their latent variables.

Finally, the measurement model’s convergent and discriminant validity were tested. Table 5 displays a matrix of the AVE, correlations and squared correlations of the variables. The AVE of the latent variables ranged between from 0.54 to 0.76, higher than the recommended cut-off value of 0.5 [54,55,56,57]. This suggests that variations in the indicators have a higher explanatory power through their latent variables rather than their errors. Next, discriminant validity was also supported because the AVE of each construct was higher than its squared correlation with other constructs.

### 4.2. Structural Model Analysis

In the first stage, the measurement model was examined for goodness-of-fit, reliability and validity. In the second stage, structural model analysis was performed to evaluate the theoretical model and its associated hypotheses. A stratified random sampling method was utilised simultaneously to minimize sampling bias. Socio-demographic variables, such as age, gender and income, were incorporated into the model to justify their effects on the adoption of SAVs. Acheampong and Cugurullo [44] stated that such socio-demographic variables can significantly affect the adoption of AVs like SAVs.

**Figure 2:**
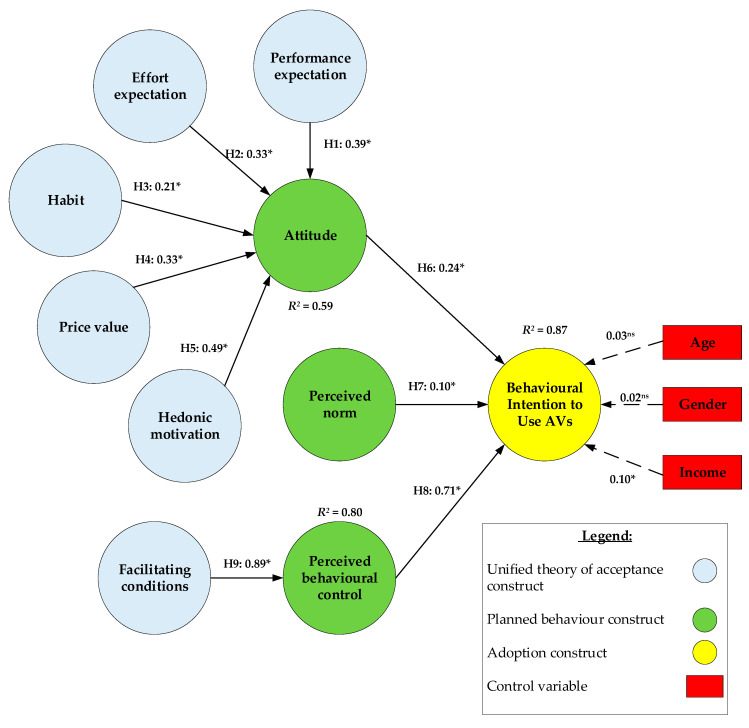
Parameter estimation of proposed model. * indicates that the path estimate is significant (*p* < 0.05); ^ns^ indicates not significant; Model fit indices: *χ^2^/df* = 2.66 (*p* < 0.05); CFI = 0.95; TLI = 0.96; RMSEA = 0.08.

As shown in Figure 2, consumer adoption of SAVs was regressed on the control variables, which include ‘age’, ‘gender’ and ‘income’. These variables are known to influence consumer adoption of SAVs in the literature [11]. Accordingly, their standardized regression estimates were 0.03, 0.02, 0.10, respectively. This indicates that only ‘income’ had a significant, positive effect on user intention to adopt SAVs. This is because individuals with a high salary are more willing to pay for novel technologies like SAVs. The effects of age’ and ‘gender’ were not significant because elderly people are not very interested in new technologies. Furthermore, prior studies have also stated that men are more likely to seek and have more interest in innovative technologies while women are likely to consider more aspects and sensitive details (i.e., price, monetary value of products, pragmatic purposes) [23]. Regardless, the effect of the socio-demographic variables was quite weak, suggesting the stronger theoretical predictors as presented in Figure 2.

The results in Figure 2 indicate that measurement model exhibits good fit (i.e., *χ^2^*/df = 2.66, *p* < 0.05, *df* = 528; CFI = 0.95; TLI = 0.96; RMSEA = 0.08) [50]. The path coefficients and hypothesis testing results are shown in each part segment in the model. In addition, the squared multiple correction (*R^2^*) of three endogenous variables were also recorded as follows: attitude (0.59), perceived behavioural control (0.80) and intention to use SAVs (0.87). All of the *R^2^* values are greater than 0.5 which highlights the level of explained variance in this structural model.

The five basic structures of the UTUAT2 namely performance expectation, effort expectation, habit, price value and hedonic motivation were confirmed. Accordingly, their effect indexes are 0.39, 0.33, 0.21, 0.33 and 0.49, respectively. This exhibited that performance expectation, effort expectation, habit, price value and hedonic motivation have positive, significant impacts on attitude toward using SAVs. Hence, H1, H2, H3, H4, H2 are accepted. Based on these dimensions, the variance explained in attitude toward using SAVs was 59% (*R^2^* = 0.59). In general, the results attributed the current study’s argument that the UTUAT2′s structures lead to the formation of users’ attitude toward using SAVs.

For instance, regarding performance expectation, enhancing the comprehensive insight of travellers about potential advantages of SAVs over traditional taxis or ride hailing services can lead to a positive attitude toward using SAVs. These SAVs’ advantages are reducing vehicle crashes, increasing traveller convenience, improving environmental utility (i.e., reduced emissions and congestion) and diminished economic burden (i.e., reduced travel costs, parking fees). Concerning effort expectation, when using SAVs is less complex and easier for users, it will require less time and effort on learning to use and interact with SAVs, thereby it also offers greater comfort for users. If consumers establish a habit using novel technology in a certain period, then they will have greater interaction and familiarity with SAVs. This will offer users better support in usage and enhance economic (i.e., learning time) and functional (i.e., diminishing effort toward learning). When consumers perceive that SAVs’ service provides better prices than conventional transport public, offers cost-efficiency (i.e., reduced trip cost, no parking prices) and the benefits and utilities that they process are greater than monetary cost, they will tend to have a positive attitude toward using SAVs. As for hedonic motivation, utilizing the innovation such as SAVs can stimulate users to seek out novel function which will in turn increase enjoyment. This may lead to a positively influence attitude.

Figure 2 also shows one of the structures of the TPB namely facilitating conditions that has positive, significant effects on perceived behavioural control (0.89). Therefore, H_8_ is accepted in this measurement model. This value explains 77% of the variance in perceived behavioural control. The presence of resources of facilitating conditions (i.e., customer service, time, money, government support) will improve SAVs’ adaptability and perform the behaviour. In addition, when travellers view SAVs to be compatible with the public transport they already used before, the behavioural control of actual usage SAVs would be shaped since using it would be easier and more comfortable.

Next, three main structures of the TPB namely attitude, subject norm and perceived behavioural control were confirmed all to have positive, significant influences on intention to use SAVs (*p* < 0.05). Hence, H_6_, H_7_, H_9_ are accepted. This result is evidence for the attitude which suggests that perception (i.e., ease of use, safety, cost saving) and emotions (i.e., pleasant, fun) about SAVs may lead travellers to choose SAVs. As for subject norm, significant referents such as family members, friends and colleagues may greatly pressure travellers to perform a particular behaviour. As for perceived behavioural control, when individuals perceive they process considerable resources which include internal factors such as skill, insight, recognition and external factors such as time, money, opportunity to make full use of SAVs, then the obstacles travellers recognize will be diminished and the greater perceived control will be shaped. Passengers will be more willing to use SAVs if they perceive SAVs is easier to use and reasonably priced than other public transportation in the same condition. The above arguments and statistical results explain for a high adoption level (89%) of the variance in user intention to use SAVs (*R^2^* = 0.87). This again endorses the strong explanation of constructs, which is comprehensively integrated into the UTUAT2 and the TPB.

## 5. Conclusions

The objective of the current study is to analyse the factors affecting user adoption of SAVs and specify their interrelationships by applying the theoretical lenses of the UTUAT2 and the TBP. Accordingly, these two theories are developed due to several reasons. Firstly, through the theoretical perspective, this study captures the essential aspects relevant concerns of customer intention to use SAVs and contributes the knowledge to their travel mode choice that significantly influent the development of the self-driving novel technology industry which includes SAVs. Secondly, by introducing and integrating five determinants of the UTUAT2 (i.e., performance expectation, effort expectation, habit, price value and hedonic motivation) and four determinants of the TBP (i.e., facilitating conditions, attitude, subject norm and perceived behavioural control), the conceptual model was established. In addition, the study indicates that the two theories are complementary and give power explanation to the conceptual model.

An online survey was conducted at Da Nang, Vietnam and completed by 268 participants. The statistical results describe an essential interrelationship between five constructs of the UTUAT2 and four constructs of the TBP which influences the adoption of SAVs. With reference to Figure 2, it is noted that perceived behavioural control has the largest direct effect on user adoption of SAVs. This is followed by attitude and perceived norm. As for the determinants of attitude, hedonic motivation has the largest effect, followed by performance expectation, effort expectation, price value and habit.

This study has contributed to the theoretical development of novel and innovative car research in several ways. In particular, this study expands the range of factors that influence the actual use of SAVs by synthesizing two theories, namely the UTAUT2 and TPB. Almost no such combination has done before in theoretical research. This synthesis has provided a more accurate prediction of consumer adoption of SAVs. Moreover, this study also contributes a deeper understanding of the interconnection between factors impacting consumer adoption of SAVs. As a result, the theoretical model provides clear analysis of factors influencing the adoption of SAVs.

The current study can provide important data for government and vehicle manufacturers to develop such novel transportation systems and encourage the adoption of SAVs. For example, they can improve the facilitating conditions to create favourable conditions for consumers to access easily and understand clearly about SAVs by providing online tutorials, test drives, advertising. They should offer preferential policy (i.e., subsidies and tax incentives) and pay attention to aesthetic, service design to make customers willing to use SAVs and enhance the level of perceived behavioural control. Next, government and vehicle manufactures can also prepare educational campaigns for benefits of SAVs can offer the public such as reducing crashes, creating cost-efficiency, diminishing traffic jam, fuel consumption and emission of greenhouse gases, enhancing convenience and enjoyment. Consumers often compare SAVs with traditional taxis, ride hailing services or prior travel modes that they had used before, thus, when enhancing the aforementioned benefits perceived, customers could have a positive attitude to SAVs and are more likely to motivated to accept such novel technology.

However, the current study still has some limitations. Firstly, the results may only be applicable to Da Nang, Vietnam. They may not apply to other countries or cultures which have their own political, environmental, social, technological, legal and economic landscape. Hence, future research can perform comparison analysis to validate the results. Secondly, SAV is an emerging innovative technology in the market and still not available to the public yet, this could limit the respondent’s understanding of such technology. Further studies could provide the respondents empirical evidence, test drives or presentations to ensure they have a clear insight about benefits and better judgment about SAVs. Thirdly, the determinants selected in this study may not capture all the determinants that could affect the adoption of SAVs in Da Nang, Vietnam. Further studies can consider new determinants such as trust, perceived risk which can influence user adoption of SAVs. Finally, future research can apply other acceptance theories such as innovation diffusion theory and trust theory to enrich their models.

## Figures and Tables

**Table 1:** Review of theories regarding users’ intention to use shared autonomous vehicles.

Theory Characteristics	Theory of Planned Behaviour (TPB)	Unified Theory of Acceptance and Use of Technology 2 (UTAUT2)
Paradigm	Psychology	Psychology and Behavioural Economics
**Basic Assumption**	The adoption of an innovative product can be affected by attitudes, control and norms	The adoption of an innovative product can be affected by dimensions relating to expectations, values, habits and enjoyment
**Representative Constructs**	Attitudes; subjective norms; perceived behavioural control	Performance expectation; effort expectation; habit; price value; hedonic motivation; social influence*; facilitating conditions
**Model Contribution**	This theory can explain how attitudinal, normative and control belief components affect the adoption of SAVs	This theory can explain how the representative constructs facilitate the formation of positive attitudes towards SAV adoption

*Note:* Social influence carries very similar meaning with subjective norms. For model parsimony, the former has been subsumed under the latter.

**Table 2:** Constructs, Response Anchors, Measures and Sources.

Constructs	Response Anchors and Measures	Adapted Sources
Performance Expectation (*PE*)	*Strongly disagree (1)/Strongly agree (5)*	[44,45]
	PE1. SAVs would enable me to save time	
	PE2. SAVs will reduce traffic congestion	
	PE3. SAVs will reduce emissions	
	PE4. Overall, SAVs is useful and advantageous	
Effort Expectation (*EP*)	*Strongly disagree (1)/Strongly agree (5)*	[44]
	EE1. Interacting with SAVs does not require a lot of mental effort	
	EE2. It will be easy for me to travel in a SAV	
Habit (*HT*)	*Strongly disagree (1)/Strongly agree (5)*	[46]
	HT1. Using SAVs would become a habit for me	
	HT2. Using SAVs would be something I do without thinking	
	HT3. Using SAVs would be a part of my daily routine	
	HT4. I would be addicted to using SAVs	
Price Value (*PV*)	*Strongly disagree (1)/Strongly agree (5)*	[46]
	PV1. I could save money by using SAVs	
	PV2. I would like to search for cheap deals in SAV services	
	PV3. SAVs would offer better value for money	
	PV4. SAVs would offer valuable promotions for me	
Facilitating Conditions (*FC*)	*Strongly disagree (1)/Strongly agree (5)*	[44,47]
	FC1. The Vietnam government is active in setting up facilities to enable SAV commerce.	
	FC2. Advances in technology will enable safer SAVs	
	FC3. SAVs would be compatible with other forms of transport I use	
	FC4. I would be able to get help from others when I have difficulties using SAVs	
Hedonic Motivation (*HM*)	*Strongly disagree (1)/Strongly agree (5)*	[47]
	HM1. Using SAVs would be fun	
	HM2. Using SAVs would be enjoyable	
	HM3. Using SAVs would be pleasant	
Attitude (*AT*)	*Strongly disagree (1)/Strongly agree (5)*	[45]
	AT1. I am excited about the possibilities offered by new technologies	
	AT2. I think advancements in technology is generally a positive thing	
Subjective Norm (*SN*)	*Strongly disagree (1)/Strongly agree (5)*	[45]
	SN1. I will travel in a SAV if my friends does the same	
	SN2. I will travel in a SAV if my family does the same	
	SN3. I will travel in a SAV if my significant references do the same	
	SN4. SAVs will be the norm on our roads in the future	
Perceived Behaviour Control (*PVC*)	*Strongly disagree (1)/Strongly agree (5)*	[19,48]
	PVC1: I would have the necessary resources, time and opportunities to use SAVs	
	PVC2: I would have the necessary knowledge to use SAVs	
	PVC3: Whether or not I use SAVs when traveling is completely up to me	
Intention to Use SAVs (*ITU*)	*Strongly disagree (1)/Strongly agree (5)*	[49]
	ITU1: I would consider using SAVs when they are available in the market	
	ITU2: I would recommend SAVs to my family and peers	
	ITU3: I would encourage others to use SAVs	

*Note:* The measures for hedonic motivation capture different aspects of users’ positive experience.

**Table 3:** Respondents’ profile.

Characteristics	Indicators	Frequency (*n* = 268)	Proportion (%)
Gender	Male	126	47
	Female	134	53
Age (years)	<18	52	19.4
	18–35	94	35.1
	36–45	67	25
	>45	55	20.5
Driving Experience	<1 year	57	21.1
	1–5 years	63	24
	6–10 years	59	21.9
	>10 years	89	33
Monthly Income (million VND) (1 USD = 23,500 VND)	<6 (<$255)	69	25.6
	6–12 ($255–$510)	91	34.4
	13–20 ($510–$850)	74	27.4
	>20 (>$850)	34	12.6

**Table 4:** Confirmatory factor analysis results.

Constructs	Indicator	λ	AVE	CR
Performance Expectation (*PE*)	PE1	0.70	0.54	0.82
	PE2	0.75		
	PE3	0.73		
	PE4	0.76		
Effort Expectation (*EE*)	EE1	0.59	0.57	0.72
	EE2	0.89		
Habit (*HT*)	HT1	0.84	0.74	0.92
	HT2	0.90		
	HT3	0.90		
	HT4	0.79		
Price Value (*PV*)	PV1	0.76	0.69	0.90
	PV2	0.84		
	PV3	0.84		
	PV4	0.85		
Hedonic Motivation (*HM*)	HM1	0.83	0.76	0.90
	HM2	0.88		
	HM3	0.89		
Facilitating Conditions (*FC*)	FC1	0.79	0.57	0.84
	FC2	0.74		
	FC3	0.72		
	FC4	0.78		
Attitude (*AT*)	AT1	0.83	0.69	0.82
	AT2	0.83		
Subjective Norm (*SN*)	SN1	0.94	0.70	0.90
	SN2	0.94		
	SN3	0.81		
	SN4	0.6		
Perceived Behavioural Control (*PVC*)	PVC1	0.83	0.54	0.81
	PVC2	0.82		
	PVC3	0.51		
Intention to Use SAVs (*ITU*)	ITU1	0.57	0.60	0.77
	ITU2	0.88		
	ITU3	0.84		

*Note:* Model fit indices: *χ^2^/df* = 2.39, (*p* < 0.05); CFI = 0.96; TLI = 0.97; RMSEA = 0.07. AVE stands for average variance extracted. CR stands for composite reliability.

**Table 5:** AVE, correlations, and squared correlations of the constructs.

	PE	EE	HT	PV	HM	FC	AT	SN	PVC	ITU
PE	0.54 ^a^	0.34 ^c^	0.28	0.25	0.31	0.34	0.21	0.08	0.22	0.27
EE	0.58 ^b^	0.57	0.37	0.22	0.31	0.37	0.14	0.14	0.35	0.28
HT	0.53	0.61	0.74	0.36	0.35	0.36	0.13	0.14	0.32	0.30
PV	0.50	0.47	0.60	0.69	0.38	0.44	0.23	0.17	0.38	0.37
HM	0.56	0.56	0.59	0.62	0.76	0.49	0.28	0.18	0.44	0.44
FC	0.58	0.61	0.60	0.66	0.70	0.57	0.31	0.23	0.40	0.42
AT	0.46	0.37	0.36	0.48	0.53	0.56	0.69	0.06	0.28	0.31
SN	0.28	0.38	0.37	0.41	0.43	0.48	0.25	0.70	0.24	0.23
PVC	0.47	0.59	0.57	0.62	0.66	0.63	0.53	0.49	0.54	0.52
ITU	0.52	0.53	0.55	0.61	0.66	0.65	0.56	0.48	0.72	0.60

*Note:*^a^ AVE values are along the main diagonal; ^b^ Correlations between constructs are below the main diagonal; ^c^ Squared correlations between constructs are above the main diagonal. See Table 4 for abbreviations of constructs.
